# Providing Belugas (*Delphinapterus leucas*) in Controlled Environments Opportunities to Thrive: Health, Self-Maintenance, Species-Specific Behavior, and Choice and Control

**DOI:** 10.3389/fpsyg.2019.01776

**Published:** 2019-08-02

**Authors:** Heather M. Hill, Hendrik Nollens

**Affiliations:** ^1^Psychology Department, St. Mary's University, San Antonio, TX, United States; ^2^SeaWorld Parks and Entertainment, Orlando, FL, United States

**Keywords:** beluga (*Delphinapterus leucas*), social composition, welfare, variability, choice

The health and welfare of animals in controlled environments continues to improve as evidence-based practices inform best care processes (Ward et al., 2018; Wolfensohn et al., 2018). Zoos and aquariums play an integral role in educating the public about Earth's fauna and flora (Maple and Perdue, 2018). Zoo and aquariums are learning environments for visitors and scientists, who come because of their appreciation for the animals and who trust that they are provided with the best possible welfare. Zoological facilities must continue to systematically and empirically measure animal welfare so science can inform decisions ensuring the optimal health and veterinary care practices along with creating environments that promote species-specific behavior and interactions, choice, and variability (Maple and Perdue, 2018; Ward et al., 2018; Wolfensohn et al., 2018). With increasing partnerships between external researchers and zoological facilities, the scientific investigation, and understanding of the components of animal welfare continues to expand (Fernandez and Timberlake, 2008; Ward et al., 2018). For example, academic-zoological partnerships have established that naturalistic exhibits can promote species-specific behaviors (e.g., Finlay et al., 1988; Ogden et al., 1990; Yilmaz et al., 2010; Chih Mun et al., 2013), and the placement of different types of enrichment promotes investigative behavior and greater habitat use (e.g., Vick et al., 2000; Clark, 2017; Makecha and Highfill, 2018). Research has also indicated that the needs of a given species and the individuals making up the facility's population must be considered when utilizing different types of enrichment, social groupings, or habitat configurations (Rose and Croft, 2015; Wolfensohn et al., 2018; Nollens et al., 2019).

The behavior and social interactions of a relatively stable white whale, or beluga (*Delphinapterus leucas*) population at a North American facility has been studied systematically since 2007. From long-term weekly documentation calf behavioral development and maternal care, behavioral milestones, such as nursing, swim positions, locomotor development, social interactions, and play behaviors have been established and validated with belugas from other facilities and in their natural habitat (Krasnova et al., 2006, 2009; Hill, 2009; Karenina et al., 2010, 2013; Hill et al., 2013, 2018a; Hill and Campbell, 2014; Hill and Ramirez, 2014). Maternal care behaviors are conserved across both habitats (Krasnova et al., 2006, 2009; Hill, 2009; Karenina et al., 2010, 2013; Hill et al., 2013, 2017), but show individual variation much like bottlenose dolphins in human care or the wild (Hill et al., 2007; Gibson and Mann, 2008; Stanton and Mann, 2012).

Results illustrated that dynamic social groupings decreased time spent swimming alone and provided opportunities for species-specific behavior, such as engaging with the environment whether it was interacting with different objects (e.g., enrichment devices, permanent fixtures, organic materials, or water) or with each other (Hill and Ramirez, 2014; Hill et al., 2015b, 2018b). Pool configuration also influenced the actions of the belugas (unpublished data). When multiple pools were available, the belugas engaged in more dynamic and variable swim patterns. Moreover, access to multiple pools provided opportunities for choice and self-maintenance: individual belugas could move out of sight of conspecifics or choose a specific area in which to swim. More recently, based on data from three different facilities, male-male interactions (whether interspecific or intraspecific) have emerged as a significant element in beluga socialization, appearing early in the juvenile years and solidifying as the males age (Glabicky et al., 2010; Hill et al., 2015a, 2018b; Mazokowski et al., 2018). These socialization patterns appear to be conserved as well based on genetic work on beluga distributions in several beluga stocks (reviewed by Colbeck et al., 2013; O'Corry-Crowe et al., 2018).

Social composition, complexity of environment through pool configurations, opportunities for intra- and inter-species interactions, and variable access to different forms of enrichment have produced measurable behavioral and physiological outcomes that are indicative of enhanced welfare of belugas in human care. These findings corroborate those found with a number of terrestrial species in zoological environments (Finlay et al., 1988; Ogden et al., 1990; Vick et al., 2000; Yilmaz et al., 2010; Chih Mun et al., 2013; Clark, 2017; Makecha and Highfill, 2018; Maple and Perdue, 2018; Wolfensohn et al., 2018). Overall, dynamic and variable experiences increased the belugas' activity (i.e., intra- and inter-species interactions, play, engagement with environmental stimuli), reduced periods of solitary swims, and provided opportunities to thrive.

The physiological health of the majority of the belugas present in the population has remained stable and strong, and as such, the population has served as a baseline reference for evaluating free-ranging belugas (Norman et al., 2012, 2013). Belugas are a species of great interest because of their vulnerability to climate change, role as a sentinel species, importance to subsistence, and for understanding threats to at-risk populations. Not only do belugas in controlled environments serve as control populations, but the beluga husbandry expertise provided from aquaria has, in part, allowed for the safe and expedient collection of blood, feces, gastric contents, skin, blubber, blowhole swabs, breath exhalations, as well as morphological, auditory, and ultrasound measurements from 56 belugas in Bristol Bay, AK. Combined, the information gained has advanced our understanding of free-ranging beluga health, physiology, disease exposure, immunology, body condition, hearing, habitat use, and the risks free-ranging belugas face.

After almost 12 years of consistent weekly observations, this academic-zoological collaboration has produced a substantial body of behavioral evidence indicating that beluga welfare is enhanced through variability and complexity with regard to social compositions, enrichment, and habitat configurations. This evidence has been further substantiated by physiological parameters that have been used as controls for assessing the health status of free-ranging belugas. However, as summarized by both Maple and Perdue (2018) and Wolfensohn et al. (2018), individual differences and preferences must be considered in any welfare assessment. Responses to enrichment attempts are variable and subject to the individuals themselves, their current motivational state, and other environmental factors that may or may not be identifiable (e.g., social status change, subclinical illness). Between learning about the needs of the animals themselves and how that knowledge might benefit both captive and wild populations, this long-term study of a reproductively active beluga population that simulates the natural composition of beluga social groupings has demonstrated that variability and choice in different aspects of the daily environment are more central to beluga welfare than the permanent habitat itself. Active collaborations between external researchers (academic researchers in this case) and zoological facilities, such as in the case of this long-term beluga behavioral study, are essential for maintaining and improving animal welfare in human care and educating current and future generations on the need to care for and conserve our planet's resources.

## Author Contributions

HH drafted the text regarding the body of behavioral research summarized in the article. HN edited and summarized the research discussing the physiological and biological contributions of research performed with belugas in controlled environments and applied to their wild counterparts.

### Conflict of Interest Statement

The authors declare that the research was conducted in the absence of any commercial or financial relationships that could be construed as a potential conflict of interest.
